# Hamstring-to-Quadriceps Ratio in Female Athletes with a Previous Hamstring Injury, Anterior Cruciate Ligament Reconstruction, and Controls

**DOI:** 10.3390/sports7100214

**Published:** 2019-09-28

**Authors:** Eleftherios Kellis, Nikiforos Galanis, Nikolaos Kofotolis

**Affiliations:** 1 Laboratory of Neuromechanics, Department of Physical Education and Sport Sciences at Serres, Aristotle University of Thessaloniki, 62100 Thessaloniki, Greece; kofotol@phed-sr.auth.gr; 2 Division of Sports Medicine, Department of Orthopaedics, General Hospital Papageorgiou, Aristotle University of Thessaloniki Medical School, 56403 Thessaloniki, Greece; ngalanismed@gmail.com

**Keywords:** isokinetic, strength balance, mixed ratio, ACL, hamstring injury

## Abstract

Muscle strength imbalances around the knee are often observed in athletes after anterior cruciate ligament (ACL) surgery and hamstring muscle injury. This study examined three hamstrings-to-quadriceps (H:Q) strength ratio types (conventional, functional, and mixed) in thirteen female athletes with a history of hamstring injury, fourteen basketball players following ACL reconstruction and 34 controls. The conventional (concentric H:Q) peak torque ratio was evaluated at 120°·s^−1^ and 240°·s^−1^. The functional (eccentric hamstring to concentric quadriceps) torque ratio was evaluated at 120°·s^−1^. Finally, the mixed (eccentric hamstrings at 30°·s^−1^ to concentric quadriceps at 240°·s^−1^) torque ratio was calculated. Both ACL and the hamstring-injured groups showed a lower quadriceps and hamstrings strength compared with controls (*p* < 0.05). However, non-significant group differences in the H:Q ratio were found (*p* > 0.05). Isokinetic assessment of muscle strength may be useful for setting appropriate targets of training programs for athletes with a history of ACL surgery or hamstring strain. However, isokinetic evaluation of the H:Q ratio is not injury—specific and it does not vary between different methods of calculating the H:Q ratio.

## 1. Introduction

Muscle strength imbalances frequently refer to the relationship between absolute muscle strength developed by antagonistic muscle groups around a joint. The hamstrings-to-quadriceps (H:Q) torque ratio takes into consideration the function of two opposing (agonist–antagonist) muscle groups and it represents the most frequently used parameter to estimate muscle strength balance [1,2,3]. A lower H:Q ratio has been implicated as a potential mechanism for various lower extremity injuries, such as anterior cruciate ligament (ACL) [4] and hamstring injuries [1,2]. For this reason, the isokinetic evaluation of H:Q torque ratio constitutes routine testing of athletes in the preseason period [1,5]. Today there are various types of ratios which can be used to assess muscle strength balance: first, the conventional ratio, representing the concentric hamstrings (H_CON_) to quadriceps (Q_CON_) torque ratio [3], second, the functional ratio, which is estimated by dividing the eccentric hamstring (H_ECC_) by the Q_CON_ torque at any given angular velocity [3,6], and, finally, the “mixed” ratio which is estimated as the ratio of H_ECC_ torque at 30°·s^−1^ to Q_CON_ torque at 240°·s^−1^ [2,6].

A systematic review has shown that that as many as half of the athletes who had undergone ACL reconstruction had not returned to the same competitive level of sport 12 months after surgery [7]. ACL reconstruction surgery, however, leads to strength deficits for both the quadriceps [8] and the hamstrings [8,9,10,11,12]. Strength deficit depends on the type of graft [12]. In particular, systematic reviews have shown that that individuals who underwent ACL reconstruction with a semitendinosus-gracilis (STG) graft reveal larger deficits in hamstring muscle strength which implies that the H:Q torque ratio (calculated using all methods) is low as long as two years post-surgery [12,13]. Nevertheless, comparison of the H:Q torque values reported for individuals who underwent ACL reconstruction differs markedly between studies. For example, a recent study [14] has shown that 12 months after ACL surgery the conventional H:Q torque ratio at 60°·s^−1^ had a value of 1.0. Other studies, however, reported a much lower conventional H:Q ratio (<0.70) for the same follow-up period and isokinetic testing condition [15]. Consequently, there is inconsistent evidence on muscle strength imbalances for athletes who returned to sport after ACL surgery. Further, although in most functional activities the hamstrings operate eccentrically [3], research has focused on the conventional H:Q ratios rather than the functional and mixed H:Q ratios.

The H:Q ratio is often considered a risk factor for hamstring muscle injury [1]. Having an incident of previous injury is probably the most significant risk factor for hamstring injury [16]. Sugiura et al. [17] reported that sprinters who sustained an injury throughout a season had an H_ECC_ strength deficit at 60°·s^−1^ but no deficits in H_CON_ and quadriceps strength compared with controls. This implies that the functional (H_ECC_/Q_CON_) torque ratio is less in players with a previous injury than controls. Others, however, have reported no differences in the conventional H:Q ratio between the injured limbs of injured subjects and the average of the two limbs of uninjured individuals [18,19]. Consequently, the issue of long-term effects of hamstring injury on H:Q ratio remains inconclusive.

By definition a low isokinetic H:Q strength ratio can be the result of low hamstring muscle strength or high quadriceps muscle strength or both. In theory, athletes who sustain a previous hamstring strain and those who underwent ACL surgery with a semitendinosus graft should display a weakness of the hamstring muscle group and hence, a low H:Q strength ratio might be expected. Since ACL reconstruction and hamstring muscle strains can lead to long-term deficits in strength, review papers have emphasized the need for further improvement in the management of such injuries and return-to-play criteria [20,21]. Early identification of H:Q strength imbalances between antagonist muscle groups may provide valuable information for the design of individualized exercise programs for those athletes who returned to sport following an ACL or hamstring injury. In addition, it is known that female athletes show a greater risk for sport injury, especially ACL injuries [22]. There is evidence that females with a lower conventional H:Q torque ratio may display a risk for ACL injury than males [5] and this may be related to a decreased ability to control knee motion in the coronal and sagittal planes [23,24]. Therefore, the purpose of this study was to examine differences between female athletes who sustained a previous hamstring injury, athletes who underwent ACL reconstruction and training-matched controls. A secondary purpose was to examine if differences between different types of injury and controls in H:Q ratio depend on the type of H:Q ratio. It was hypothesized that injured athletes would display a lower H:Q ratio than controls and that group differences in H:Q ratio would differ between conventional, functional and mixed H:Q ratios.

## 2. Materials and Methods

### 2.1. Participants

A total of 172 female athletes (88 sprinters and hurdlers and 84 basketball players) from track-and-field and various basketball clubs were initially invited to participate after signing informed consent forms. All participants trained at least three times a week for the past five years prior to the study and volunteered to participate in this study after signing written informed consent. The necessary sample size to achieve statistical power was calculated using G-power (v.3.1.9.4, University Kiel, Kiel, Germany). For a multivariate analysis of variance (MANOVA) global effect on H:Q ratios, the following inputs were provided: effect size = 0.25, power = 0.80, type 1 error = 5%. This model yielded a sample size of 13 participants per group. For univariate group comparisons, a minimum sample size of 14 participants per group was estimated.

At the initial contact, 27 athletes reported a hamstring injury within the past year. Similarly, 32 athletes reported an ACL-reconstruction surgery and returned to sport within the year. To be included in the hamstring-injured group, the following criteria were applied, (a) a sudden onset of nonimpact posterior thigh pain in training or games within the previous 12–16 months, (b) injury of moderate intensity with structural damage identified using MRI or ultrasound and classified by a health professional [25], (c) a minimum of 14 days lost of regular training due to injury and (d) successful return to sport for at least six months prior to this study. To be included in the ACL group, the criteria were: (a) An isolated ACL rupture with absence of any injury of other structures, (b) unilateral ACL reconstruction with a STG graft, (c) surgery occurred 7–12 months prior to this study, (d) followed the same postoperative rehabilitation by the same medical team and (e) successful return to sport for at least six months prior to this study. Participants were excluded if they experienced the same injury before, they had any pathology or surgery in the hips, ankles, or feet; any neurologic, cardiovascular, metabolic, rheumatic, or vestibular disease.

Based on their self-report and a clinical examination by a health professional, of the 27 athletes with a previous hamstring injury, 14 were excluded, including seven athletes because they had both a hamstring and a ligamentous injury, four because injury occurred less than six months prior to the study and three because injury led to less than 14 days loss of training. Similarly, of the 32 players with ACL reconstruction surgery, 18 were excluded because they had other secondary injuries. Of the remaining participants, those who did not experience any musculoskeletal, neuromuscular or other type of injury or condition that led to absence from training within the past two years were invited to serve as a control group. Of these, 41 did not meet the inclusion criteria while 38 participants did not participate. Based on the above, three groups were formulated: The hamstrings-injured group (HIG) consisting of 13 females, the ACL group consisting of 14 females and the control group (CG) consisting of 34 females. The study was approved by the local Ethics Committee and all participants gave their written consent prior to participation.

### 2.2. Procedures

Maximum strength tests of the injured (ACL and HIG groups) or right (CG) limb were performed on a Humac Norm isokinetic dynamometer (Humac Norm, Cybex CSMI, Stoughton, MA, USA). The protocol included maximum strength testing of the knee flexors and extensors of the right limb from the seated position. The thigh, pelvis, and trunk were stabilized with straps. The axis of knee rotation was aligned with the lateral femoral condyle. The knee range of motion was set from 0 (full extension) to 90° of knee flexion. Prior to the test, the limb was weighed at 30° using the static gravitational correction procedure recommended by the manufacturer. First, the participants were given a familiarization session by performing five submaximal efforts at 30°·s^−1^. The main testing protocol included: five concentric knee extension and flexion repetitions at 240°·s^−1^, 5 eccentric efforts of the knee flexors at 30°·s^−1^ and five concentric and eccentric efforts of both muscle groups at an intermediate velocity of 120°·s^−1^. The sequence of tests was randomized across types of muscle action and angular speeds. The participants were instructed to exert maximal effort through the whole range of motion during the test. Between the sets, there was a time interval of one minute to minimize any fatigue effect.

The repetition with the gravity-corrected maximum joint torque was analyzed from the five repetitions. The range of motion used for analysis was restricted from 10° to 70° to avoid inertial effects. For each testing condition, the maximum torque was used for further analysis. In a pilot study, nine participants (three members from each group) were re-tested one week after to establish the reliability of the protocol. The intraclass correlation coefficient values (ICC) for the peak torques were as follows: 0.89 and 0.91 for the Q_CON_ and H_CON_ at 240°·s^−1^, respectively; 0.94 for the H_ECC_ torque at 30°·s^−1^, 0.96 and 0.97 for the Q_CON_ and H_CON_ torque at 120°·s^−1^, respectively and finally, 0.91 and 0.92 for the Q_ECC_ and H_ECC_ torque at 120°·s^−1^, respectively. Subsequently, the conventional ratios at 120°·s^−1^ (H_CON120_/Q_CON120_) and 240°·s^−1^ (H_CON240_/Q_CON240_), the functional ratio at 120°·s^−1^ (H_ECC120_/Q_CON120_) and the mixed ratio (H_ECC30_/Q_CON240_) were calculated.

### 2.3. Statistical Analyses

The statistical package for social sciences (SPSS version 25, IBM Corp., Armonk, NY, USA) was used for statistical analysis. Group differences in demographic characteristics were examined using one-way analysis of variance tests. Data were checked for normality using the Kolmogov–Smirnov test. Further, application of Levene’s test for homogeneity of variances for each dependent variable showed that the equal variances assumption for applying analysis of variance (ANOVA) statistics was met. A multivariate analysis of variance (MANOVA) design was implemented to compare various types of H:Q ratios using body mass and height as co-variates. A separate MANOVA was applied for peak torque values. If significant, univariate one-way analyses of co-variance (ANCOVAs) followed by post-hoc Tukey tests were used to examine differences between pairs of means. Effect sizes (η^2^) and statistical power were also monitored. From the MANOVA results, the mean, standard error and 95% confidence intervals of the differences in peak torque between the control group and each of the ACL and the HIG groups were also used for further analysis. Subsequently, these differences were then expressed as a percentage of the CG group mean, to provide an indication of any deficit of each group relative to the CG group. The level of significance was set at *p* < 0.05.

## 3. Results

### 3.1. Demographic Characteristics

The demographic characteristics of the three groups are displayed in Table 1. The ANOVA indicated non-significant group differences in age (*p* > 0.05). However, the participants of the ACL group were taller and heavier than the remainder groups (*p* < 0.05).

### 3.2. Peak Torque

**Figure 1:**
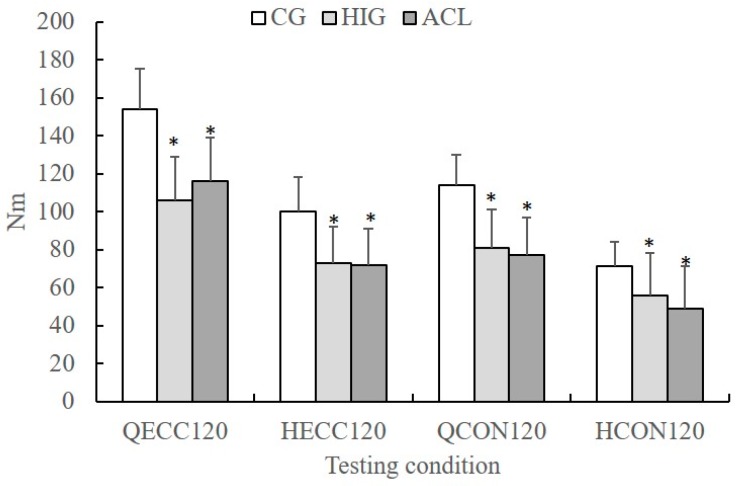
Mean group values of concentric (H_CON120_) and eccentric (H_ECC120_) hamstrings and concentric (Q_CON120_) and eccentric (Q_ECC120_) quadriceps peak torque at an angular velocity of 120°·s^−1^ for the control group (CG), the ACL reconstruction group (ACL), and the hamstring injured group (HIG) (error bars indicate standard deviation, * significant different compared with the GG, *p* < 0.05).

The MANOVA showed that there was a group difference in peak values (*p* < 0.05, η^2^ = 0.36, power = 0.99). The ANCOVA designs for each angular velocity condition showed a group effect on peak torque values (*p* < 0.05, η^2^ ranged from 0.23 to 0.49, power ranged from 0.87 to 0.99). The peak torque values recorded at 120°·s^−1^ are presented in Figure 1. In each testing condition, post-hoc Tukey tests showed that peak torque was lower in the ACL and HIG groups compared with the CG (*p* < 0.05). No differences between ACL and HIG were observed (*p* > 0.05).

**Figure 2:**
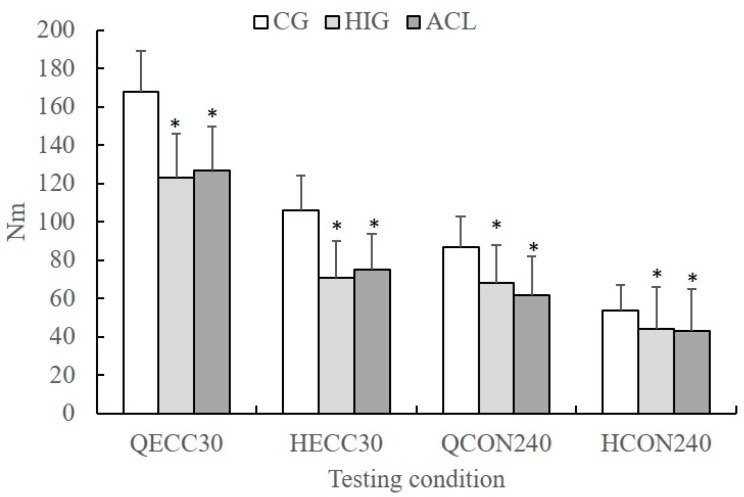
Mean group values of eccentric quadriceps (Q_ECC30_) and hamstrings (H_ECC30_) peak torque at 30°·s^−1^ and concentric quadriceps (Q_CON240_) and hamstrings (H_CON240_) peak torque at 240°·s^−1^ for the control group (CG), the ACL reconstruction group (ACL), and the hamstring injured group (HIG) (error bars indicate standard deviation, * significant different compared with the GG, *p* < 0.05).

The peak torque values recorded at 30 and 240°·s^−1^ are presented in Figure 2. Post-hoc Tukey tests showed that in all conditions, peak torque was lower in the ACL and HIG compared with CG (*p* < 0.05). To better illustrate the Group differences in peak torque values, the percentage differences between CG and ACL and between CG and HIG groups are presented in Table 2. The ACL group showed a deficit ranging from 24.4% to 36.3% in peak torque compared the CG. The HIG also showed a strength deficit compared with the CG ranging from 18.3% to 35.5%.

### 3.3. H:Q Torque Ratios

The H:Q ratios for all groups are presented in Table 3. The MANOVA designs showed that there was a non-significant group effect on H:Q ratios (*p* > 0.05, η^2^ = 0.16, power = 0.88).

## 4. Discussion

The main findings of this study were that female sprinters with a history of previous injury and basketball players with a history of ACL reconstruction showed similar H:Q ratios compared with controls. Group differences did not vary between conventional, functional and mixed H:Q ratios. In contrast, both groups showed significant strength deficits compared with the uninjured. To our knowledge, this is the first study that compares the isokinetic H:Q ratio between the two types of injury in female athletes.

The female basketball players with ACL reconstructed knees showed similar H:Q ratio with controls and HIG (Table 3). Almost one-year after ACL surgery, the basketball players showed approximately 24–36% lower (compared to CG) quadriceps and hamstrings isokinetic torque at slow and intermediate angular velocities (Table 2). This confirms previous research which concluded that most athletes who had undergone ACL reconstruction had not returned to the same competitive level of sport 12 months after surgery [7]. In our study, the conventional H:Q ratio at 120°·s^−1^ was approximately 0.60 which is similar to that reported by Fischer et al. [15] (0.67) but it is much lower than that reported by Martin-Alguacil et al. [14] (1.0). Such large differences may indicate that H:Q values may be specific to the methods used, the type of athletes and, perhaps, the rehabilitation protocol followed after ACL surgery. Our results cannot be directly compared with previous studies [12,13] as we calculated the strength deficit between injured and uninjured athletes, instead of the interlimb difference. Nevertheless, the present results are in line with previous review and metanalysis studies which reported that individuals who underwent ACL reconstruction with a STG graft displayed similar deficits in the quadriceps and hamstrings muscle strength two years post-surgery [12,13]. Quadricep deficits tend to be greater one year after surgery, but the range of deficit varies significantly between muscle groups and experimental studies [12,13]. Hiemstra et al. [26] reported that individuals who underwent ACL reconstruction with a hamstrings graft displayed a 25% deficit in concentric and eccentric hamstring and quadriceps strength compared with controls. The existence of strength deficits after ACL reconstruction has been attributed to alteration of motor unit activation and afferent input due to the ligament rupture, surgery itself, and persistent pain and effusion [11]. Muscle atrophy has been reported for the quadriceps [27,28] and the hamstrings [29] following ACL reconstruction surgery. Based on our findings, it appears athletes who sustained ACL surgery with a STG graft are expected to show similar isokinetic H:Q ratio (irrespective of calculation method) to controls, one year after surgery.

The absence of differences in the conventional, the functional and the mixed H:Q ratio between female sprinters with a history of hamstring injury and controls (Table 3) does not imply that the two groups of athletes achieved similar isokinetic peak torque. In fact, our results indicate that previously injured female sprinters showed consistently lower (~18–35%) quadriceps and hamstring peak torques at all but one testing conditions (Table 2). It should be noted that there was a tendency for a lower mixed H:Q ratio in the HIG group compared to the other two groups (Table 3) as the HIG group showed a greater strength deficit at 240°·s^−1^ compared to the deficit observed at 30°·s^−1^ (Table 2). To our knowledge, very few studies compared athletes with a previously injured hamstring muscle and controls; instead most literature examined the bilateral limb differences in the functional H:Q ratio and hamstring muscle strength [6,18,19]. Sugiura et al. [17] reported that sprinters who sustained an injury throughout a season had an H_ECC_ strength deficit at 60°·s^−1^ but no deficits in H_CON_ and Q_CON_ strength compared with controls were found. This implies that the functional torque ratio is less in players with a previous hamstring injury than controls. Using a prospective experimental design, Croisier et al. [1] showed that athletes with a history of hamstring injury showed strength deficits in both quadriceps and hamstrings, which is line with the present study. In addition, they observed that injured athletes showed a much greater deficit in H_ECC_ torque at 30°·s^−1^ than that observed for Q_CON_ torque at 240°·s^−1^. Other studies showed a similar mixed ratio (ranging from 1.29 to 1.33) of the players who sustained a hamstring injury and those players who were not injured [2,30]. All these prospective studies, however, indicated that selected players with certain cut-off values in the H:Q ratio have greater injury risk [1,2,30]. There are various factors that may have contributed to the observed strength deficiency of the HIG group. First, in our study, athletes were included when they lost a minimum of 14 days of training and games following injury. This indicates that low intensity episodes of hamstring pain or mild strains were not examined. Second, the presence of trauma after injury may have negative effects on strength [31]. Third, muscle atrophy occurs in both limbs after injury and it may spread to other muscles [32]. Finally, a neural inhibition of the injured hamstring has been observed [33], but this cannot justify the reduction in quadriceps strength after hamstring injury. Post-traumatic pain may be associated with an adaptation in motor behavior to protect the muscle from further pain or injury [34] and this may be related to long-term deficits in quadriceps strength.

It was interesting that the ACL and HIG groups showed similar H:Q ratio values (Table 3). The ACL group showed almost equal deficit in quadriceps and hamstring strength while the HIG group tended to have a greater deficit in hamstring strength (Table 2). To our knowledge, there are no studies that have directly compared muscle strength balances between athletes who experience these two different types of injury. Comparison of peak torque values between the two injured groups is difficult as any differences between sports are being masked by the long-term effects of injury as well as post-injury rehabilitation and regular training. In both cases, a large long-term deficit in the quadriceps muscle was observed even though the quadriceps muscle was not either injured (for the HIG group) or operated (in the ACL group). It should be noted that no group differences in strength were observed even when body mass and height were used as co-variates (Table 1). Furthermore, by comparison to the control group, we were unable to identify any systematic difference in strength that appeared in the HIG group and not in the ACL group and vice versa (Table 2).

Our hypothesis that the method used to calculate the H:Q ratio would influence group comparisons in isokinetic muscle imbalances was not confirmed. Although the use of functional and the mixed H:Q ratio takes into consideration the type of contraction displayed by the antagonistic muscle groups, none of these ratios, could detect any strength imbalances that are not detected by the traditional conventional H:Q ratio. The use of the mixed H:Q ratio, which is a combination of eccentric slow speed strength of the hamstrings and concentric fast speed strength of the quadriceps did not reveal any strength deficits that are either muscle-specific, speed-specific, or contraction type-specific. It is worth noting, however, that the mixed H:Q ratio was almost 15–17% less (although non-significant) in the HIG group compared with the other two groups. The absence of statistically significance can be attributed to the variability of the H:Q ratio values, which incorporates the variability of torque values, as well as the relatively small sample size, per group. As far as the conventional H:Q ratio is concerned, all groups examined in the present study display a mean ratio of 0.60 which is generally considered as “normal” [3]. It has been found that athletes with a conventional H:Q ratio less than 0.60 may be at greater risk to sustain hamstring injury [2]. Similarly, athletes with a ratio less than 0.75 display greater risk for a lower extremity injury [5] while a mixed ratio of less than 0.80 is considered as being predictive of hamstring injury [2]. It may be, therefore, suggested that such isokinetic testing protocols which allow evaluation of the various types of H:Q ratio are useful for screening players with certain cut-off values [1,2,30].

The results have some implications for routine isokinetic testing of athletes especially prior to the beginning of the game season. First, it is worth noting that athletes who sustained two significant sport injuries (ACL and hamstring strain) continue to display important weakness in strength of two major leg muscle groups of the knee long after their return to play. In this respect, such players should undergo pre-season isokinetic evaluation and, if necessary, to follow individualized exercise programs to reduce these strength deficits. Second, our results indicated that athletes with a history of injury showed a generalized knee extensor and flexor weakness and, therefore, the H:Q ratios appeared as being no different compared with uninjured players. Third, it appears that information about isokinetic muscle imbalances did not differ between conventional, functional and mixed H:Q ratios. Hence, the use of isokinetic testing protocols which include evaluation of speed-specific, contraction-type-specific, and muscle-specific agonist versus antagonist strength imbalances is not supported.

In the present study, we compared three groups of individuals, a group with unilateral ACL reconstruction, a group with hamstring injury and a control group. A limitation of this study was that isokinetic strength evaluation was not performed in both limbs. However, previous studies have shown that ACL reconstruction affects muscle strength of the contralateral limb [11] and, making the non-injured leg sub-optimal as a ‘‘healthy’’ reference. The results are also limited by the small sample size, but this is partly due to the specific inclusion criteria set for participation in this experiment. For the same reason, it has been difficult to match the three groups based on demographic characteristics. Although the groups had similar age, the ACL group participants were taller and heavier than the other groups. For this reason, group comparisons were adjusted for body mass and height effects. Nevertheless, the reader should be aware that this is not a matched case-control study.

## 5. Conclusions

Evaluation of H:Q ratios did not reveal significant long-term impairments in balance between agonist and antagonist muscle strength after hamstring injury or ACL reconstruction surgery. This was observed for conventional, functional and the mixed H:Q ratio. Athletes with a history of previous ACL surgery or hamstring injury displayed significant isokinetic strength deficits of the knee extensor and flexors compared with uninjured athletes.

## Figures and Tables

**Table 1:** Mean (± SD) age and anthropometric characteristics of the control group (CG), the anterior cruciate ligament (ACL) and the hamstring–injured group (HIG).

Variable	CG (N = 34)	ACL (N = 14)	HIG (N = 13)
Age (years)	21.5 ± 2.5	21.9 ± 2.1	20.9 ± 1.7
Height (cm)	176.4 ± 4.4	181.0 ± 4.9 *	173.9 ± 3.7
Body mass (kg)	77.0 ± 3.8	81.3 ± 3.9 *	76.4 ± 3.9

* significantly different compared with the other groups, *p* < 0.05.

**Table 2:** Mean, standard error and 95% confidence intervals of the differences in each dependent variable between the control group and each of the ACL reconstruction group (ACL) and the hamstring injured group (HIG).

Deficit	Mean Percentage Difference (95% Confidence Interval)	
Test Condition	ACL	HIG
Q_CON120_	35.7 ± 7.5 (14.9–50.8)	28.6 ± 7.3 (11.2–46.1)
H_CON120_	36.3 ± 9.6 (8.4–52.1)	21.1 ± 8.7 (0.1–42.3)
Q_ECC120_	24.4 ± 9.1 (2.2–46.6)	30.7 ± 8.9 (9.2–52.3)
H_ECC120_	26.8 ± 9.9 (2.7–51.2)	27.9 ± 9.8 (3.5–50.7)
Q_ECC30_	25.2 ± 8.2 (4.5–45.8)	27.0 ± 8.5 (7.0–47.2)
H_ECC30_	29.7 ± 8.5 (7.7–51.7)	35.5 ± 8.8 (12.0–54.2)
Q_CON240_	33.5 ± 8.9 (7.2–50.2)	21.4 ± 8.6 (1.4–43.1)
H_CON240_	33.9 ± 10.1 (−2.1–46.2)	18.3 ± 9.3 (−3.8–43.1)

Q_CON120_, H_CON120_ = concentric peak torque of quadriceps and hamstrings at 120°·s^−1^, Q_ECC120,_ H_ECC120_ = eccentric peak torque at 120°·s^−1^, Q_ECC30,_ H_ECC30_ = eccentric peak torque at 30°·s^−1^, Q_CON240_, H_CON240_ = concentric peak torque at 240°·s^−1^.

**Table 3:** Mean (± SD) group hamstrings to quadriceps peak torque ratio for the control group (CG), the ACL reconstruction group (ACL) and the hamstring–injured group (HIG). The conventional ratios were calculated at concentric angular velocities of 120°·s^−1^.

Type of Ratio	CG (N = 34)	ACL (N = 14)	HIG (N = 13)
Conventional			
H_CON120_/Q_CON120_	0.62 ± 0.11	0.64 ± 0.09	0.68 ± 0.12
H_CON240_/Q_CON240_	0.63 ± 0.16	0.65 ± 0.07	0.69 ± 0.08
Functional			
H_ECC120_/Q_ECC120_	0.88 ± 0.22	0.94 ± 0.17	0.99 ± 0.22
Mixed			
H_ECC30_/Q_CON240_	1.24 ± 0.23	1.25 ± 0.29	1.06 ± 0.11

H_CON120_/Q_CON120_) and 240°·s^−1^ (H_CON240_/Q_CON240_). The functional (H_ECC240_/Q_CON120_) was calculated at an angular velocity of 120°·s^−1^. Finally, the mixed ratio was estimated as the eccentric hamstring torque at 30°·s^−1^ to concentric quadriceps torque at 24°·s^−1^ (H_ECC30_/Q_CON240_).
